# Evaluation of Clinical and Paraclinical Findings for the Differential Diagnosis of Autoimmune and Infectious Encephalitis

**DOI:** 10.3389/fneur.2018.00434

**Published:** 2018-06-08

**Authors:** Judith N. Wagner, Ognian Kalev, Michael Sonnberger, Ingomar Krehan, Tim J. von Oertzen

**Affiliations:** ^1^Department of Neurology 1, Kepler University Hospital, Linz, Austria; ^2^Department of Neuropathology, Kepler University Hospital, Linz, Austria; ^3^Department of Neuroradiology, Kepler University Hospital, Linz, Austria; ^4^Department of Neurology 2, Kepler University Hospital, Linz, Austria

**Keywords:** encephalitis, autoimmune disease, limbic encephalitis, neuroinfectiology, neuroimmunology

## Abstract

**Background:** The differential diagnosis of autoimmune and infectious encephalitis is notoriously difficult. For this study, we compare the presenting clinical symptoms and paraclinical test results of autoimmune and infectious encephalitis patients. A clinical algorithm for the diagnosis of autoimmune encephalitis has recently been published. We test these Graus criteria on our cohort for diagnostic sensitivity and specificity within the first week of presentation.

**Methods:** We included all patients seen at our department within a 10-year-period who were diagnosed with encephalitis. The discharge diagnoses served as the reference standard for testing the clinical algorithm for two conditions: use of all the clinical information available on a patient during the first week of hospital admission assuming undefined autoantibody status and microbiological test results (C1) vs. consideration of all the information available on a patient, including the results of serological and microbiological testing (C2).

**Results:** Eighty-four patients (33 autoimmune, 51 infectious encephalitis) were included in the study. Fifty-one (17 autoimmune, 34 infectious) had a definite clinical diagnosis. The two groups differed significantly for the presence of headache, fever, epileptic seizures, and CSF cell-count at presentation. Application of the clinical algorithm resulted in a low sensitivity (58%) and very low specificity (8%) for the diagnosis of possible autoimmune encephalitis. The latter increased considerably in the subgroups of probable and definite autoimmune encephalitis. Whereas the sensitivity of the individual diagnostic categories was clearly time-dependent, the specificity rested foremost on the knowledge of the results of microbiological testing. Anti-CASPR2- and -LGI1-associated autoimmune encephalitis and tick-borne virus encephalitis presented particular diagnostic pitfalls.

**Conclusions:** We define clinical symptoms and paraclinical test results which prove valuable for the differentiation between infectious and autoimmune encephalitis. Sensitivity and specificity of the clinical algorithm clearly depended on the amount of time passed after hospital admission and knowledge of microbiological test results. Accepting this limitation for the acute setting, the algorithm remains a valuable diagnostic aid for antibody-negative autoimmune encephalitis or in resource-poor settings. The initiation of immune therapy however should not be delayed if an autoimmune etiology is considered likely, even if the diagnostic criteria of the algorithm are not (yet) fulfilled.

## Introduction

Encephalitis is an inflammatory process affecting the cerebral parenchyma. It is associated with considerable morbidity and mortality, causing focal neurological deficits, cognitive and neuropsychiatric defects, and epilepsy (1–6). The etiology can be infectious (most often viral) or autoimmune. This field has been a very dynamic one during the last decade due to the rapidly expanding spectrum of antibodies causing autoimmune encephalitis [AE; (7)] as well as to the discovery of new infectious agents or redistribution of the geographic range of known pathogens (8). The diagnosis of AE is frequently difficult as the paraclinical testing is often unremarkable: the rate of false negative magnetic resonance imaging (MRI) and cerebrospinal fluid (CSF) analysis is particularly high in the elderly with antibodies against CASPR2 and LGI1 (9). An abnormal MRI has been described in only 30% of patients with anti-NMDA-receptor-encephalitis (NMDARE) and with CASPR2-associated AE, an abnormal CSF in 20–40% of AE patients with CASPR2- and LGI1-antibodies (10). Furthermore, about 50% of all AE patients are antibody-negative (11). But unremarkable cerebral imaging and CSF analysis may also occur in infectious encephalitis (IE), particularly in the immunocompromised (12, 13). Despite of advanced molecular and serological diagnostic techniques, the causative pathogen cannot be detected in up to 60% (14). Hence, the etiology of encephalitis remains unresolved in ~50% of all cases (15, 16).

This poses a significant dilemma as AE and IE require opposite therapeutic strategies and the early institution of therapy is associated with a more favorable outcome (17–21). In a position paper published in 2016, Graus et al. acknowledge the importance of enabling the clinician to define an early diagnosis and ground it on clinical symptoms at the time of presentation and standard paraclinical tests that are readily available (22). The authors developed an algorithm allowing for a diagnosis of probable or even definite AE solely on the grounds of clinical presentation, MRI, CSF analysis, and EEG. The aim of this paper is to test the sensitivity and specificity of this algorithm on our cohort of encephalitis patients. In particular, we aim to elucidate whether it is helpful in distinguishing AE and IE during the early stage (i.e., first week) of hospital admission. We also compare the prevalence of individual presenting symptoms and results of paraclinical tests between the two etiological groups so as to define additional markers distinguishing between them early in the course of the disease.

## Methods

### Patients

We included all patients seen at our department from 2007 to 2017 who were diagnosed with a recognized sero-clinical encephalitic syndrome (such as brachiofacial dystonic seizures with detection of LGI1-antibodies) or fulfilled the criteria of the Consensus Statement of the International Encephalitis Consortium (23) for possible, probable or confirmed encephalitis:

- Major Criterion (required):

Patients presenting to medical attention with altered mental status (defined as decreased or altered level of consciousness, lethargy or personality change) lasting ≥24 h with no alternative cause identified.

- Minor Criteria (2 required for possible encephalitis; ≥3 required for probable or confirmed encephalitis):
  - Documented fever ≥38°C (100.4°F) within the 72 h before or after presentation
  - Generalized or partial seizures not fully attributable to a preexisting seizure disorder
  - New onset of focal neurologic findings
  - CSF WBC count ≥5/cubic mm
  - Abnormality of brain parenchyma on neuroimaging suggestive of encephalitis that is either new from prior studies or appears acute in onset
  - Abnormality on electroencephalography that is consistent with encephalitis and not attributable to another cause

All patient records at our department were reviewed by an experienced neurologist (JW). The relevant information was extracted from our electronic clinical information system. Patients were included in the study if they had

- The diagnosis of definite^c^ IE or AE: defined by detection of the causative pathogen/ antibody in a patient with an appropriate clinical picture OR
- The diagnosis of a probable^c^ infectious or autoimmune encephalitis: defined by a typical clinical course—i.e., monophasic <4 weeks ± prodromal symptoms in infectious and polyphasic/undulating/monophasic >4 weeks in autoimmune encephalitis and/or a clear response to either antimicrobial or immunosuppressive therapeutic agents

Qualifiers such as “probable,” “possible,” “definite” are specified with a superscript “c” if they refer to the clinical criteria delineated above and with “a” if they refer to the algorithm by Graus et al.

Patients were excluded if they had a purulent encephalomeningitis, if the diagnosis did not meet the level of certainty specified above or if the diagnosis made after reviewing the entirety of a patient's records differed from the initial diagnosis at discharge.

All analyses were performed separately on the group of patients with a definite^c^ diagnosis as well as on the entire cohort (probable^c^ + definite^c^). Unless otherwise specified, results pertain to the former group.

### Study definitions

The confirmed discharge diagnosis was used as the reference against which the clinical algorithm was tested. We defined two conditions:

- Condition C1: the clinical information available on a patient during week 1 of his or her hospital admission was considered; their autoantibody status and the results of specific microbiological tests were assumed to be unknown
- Condition C2: all the information available on a patient was considered, including the results of serological and microbiological testing

T0 was defined as the time of onset of symptoms as reported by the patient or his family. T1 was the time of admission to our hospital. In case the patient was transferred from another hospital and all the information was available to us, we would consider T1 to refer to the external admission.

### Diagnostics

Autoantibody testing was performed using immunofluorescence and line blotting for intracellular antibodies and immunofluorescence on commercially available cell-based assays for extracellular antibodies. Immunofluorescence was carried out on EUROIMMUN tissue biochips for paraneoplastic neuronal antibodies and EUROIMMUN biochips with transfected cells for antibodies against neuronal receptors. Antibodies against intracellular antigens were also tested with EUROIMMUN line blot. For Ganglioside IgG- and IgM-antibodies detection the “Buhlmann GanglioCombi” enzyme-linked immunosorbent assay was used. Standard laboratory procedures were followed according to the manufacturer's instructions. The assays were evaluated by experienced neuropathologists. The diagnostic panels represented the standard selection of antigens described at the respective points in time (for details see Supplementary Table 1). Currently, immunological testing comprises antibodies against Hu, Yo, Ri, PNMA2 (Ma2/ta), CV2, amphiphysin, PCA2, TR, SOX1, Zic4, Recoverin, GAD, Myelin, Titin, MAG, GM1, GM2, GD1a, GD1b, GQ1b, and anti-glial nuclear antibodies as well as NMDAR-/CASPR2-/GABA B-/LGI1-/AMPA-GluR1/2- and DPPX-antibodies. Antibody screening was performed in all patients discharged with the diagnosis of AE and in 11 patients with the final diagnosis of IE. Standard microbiological screening comprised PCR and/or serology for herpes simplex virus type 1 and 2 (HSV1, HSV2), cytomegalovirus (CMV), varicella zoster virus (VZV), Epstein-Barr virus (EBV), tick-borne encephalitis virus (TBEV), borrelia and cultures for bacteria and mycobacteria (CSF and serum). It was performed in all IE cases and in all but two patients with a diagnosis of AE. Further microbiological testing was guided by clinical judgement. MRI, EEG and CSF analyses were performed according to standard protocols.

### Statistical analysis

Excel and MedCalc statistical software were used for evaluation of patient data. We calculated absolute frequencies and percentages for categorical variables and the median and range for continuous variables. Sensitivity, specificity, positive predictive value (PPV), and negative predictive value (NPV) were calculated for different categories defined by Graus et al. using our confirmed discharge diagnoses as a reference standard. For further sensitivity and specificity analyses, a receiver operating characteristic (ROC) curve analysis was performed. Groups were compared using the Chi-Square- and Mann–Whitney-*U*-Test. Statistical significance was assumed for *p* < 0.05.

The study was approved by the ethics committee of Upper Austria.

## Results

Eighty-four (44 male) patients seen in our department between January 2007 and December 2017 fulfilled the inclusion criteria. In 71 patients the discharge diagnosis was made before the publication of the diagnostic algorithm by Graus et al. making a bias unlikely. Thirty-three were diagnosed with autoimmune encephalitis (17 definite^c^ AE), 51 with infectious encephalitis (34 definite^c^ IE). Diagnoses in antibody negative AE included parainfectious AE/ADEM (3), Bickerstaff encephalitis (2), and seronegative limbic/autoimmune encephalitis (11).

### Epidemiological and clinical data for the entire (probable^c^ + definite^c^) cohort

Median age was 58 years (AE; range 13–87) and 57 years (IE; range 14–83), respectively. Median time lapse between T0 and T1 was 5 days (range 1–270) for AE and 3 days (range 1–100) for IE, median time to last follow-up defined as lapse between T1 and the last time the patient was seen at our department for any reason was 427 (range 5–2,364) for AE and 44 days (range 3–2,510) for IE. Eight (AE) and 2 (IE) patients were diagnosed with neoplastic disease: 1 patient each with Non-Hodgkin Lymphoma and chronic lymphatic leukemia in IE and cancer of unknown primary (2 patients), ovarial teratoma (2 patients), pulmonary adenocarcinoma (1 patient), pulmonary neuroendocrine tumor (1 patient), mesothelioma (1 patient), and prostate carcinoma (1 patient) in AE. A subset of our AE patients have been described before (24, 25).

### Epidemiological and clinical data for the definite^c^ cohort

This cohort included 51 patients (34 IE; 26 male). Median age was 57 years (AE; range 13–73) and 61 years (IE; range 16–77), respectively. Median time lapse between T0 and T1 was 10 days (range 1–270) for AE and 4 days (range 1–100) for IE, median time to last follow-up defined as lapse between T1 and the last time the patient was seen at our department for any reason was 920 (range 46–2,364) for AE and 58 days (range 3–2,510) for IE. Baseline characteristics of the two diagnostic groups are summarized in Table 1, the microorganisms and autoantibodies detected in those patients with definite IE/AE are listed in Tables 2A,B.

**Table 1:** Characteristics of patients (definite^c^) contained in the two diagnostic groups of autoimmune (AE) and infectious (IE) encephalitis.

**Diagnostic group**	**Total (male)**	**Age (median, range)**	**Days T0 to T1 (median, range)**	**Days—follow-up (median, range)**	**Tumor *n* (%)**
Autoimmune encephalitis	17 (9)	57 (13;73)	10 (1;270)	920 (46;2364)	6 (35)
Infectious encephalitis	34 (17)	61 (17;77)	4 (1;100)	58 (3;2510)	0 (0)

*T0 specifies the time of symptom onset, T1 the time of hospital admission*.

**Table 2A:** Antibodies detected in patients with definite^c^ AE.

**Antibody**	***n***
NMDAR	5
LGI1	4
CASPR2	3
Ma2	2
Ri	1
GabaB	1
SOX1^*^	1
Amphiphysin^*^	1

*The antibodies marked with an asterisk were found in the same patient*.

**Table 2B:** Microorganisms detected in patients with definite^c^ IE.

**Microorganism**	***n***
Tick-borne encephalitis virus	25
Herpes simplex virus type 1	5
Ebstein-Barr virus	2
Tropheryma whippelii	1
Varicella zoster virus	1

### Signs and symptoms (condition C1; definite^c^)

The majority of patients with IE presented with fever (94%), headache (56%), quantitative alterations of consciousness (56%), and psychiatric symptoms (including personality changes and psychomotor retardation; 82%). Speech disorders (24%), focal neurological deficits (29%), and epileptic seizures (21%) were frequently encountered as well. Focal neurological signs in IE comprised central paresis (4 patients), cerebellar symptoms (3 patients), cranial nerve palsies (4 patients) and oculomotor system disturbances (3 patients; some patients displayed two or more focal neurological symptoms).

In AE, headache, fever, alterations of consciousness and psychiatric symptoms were significantly less prevalent at presentation (0, 12, 12, and 47%, respectively). Epileptic seizures were frequently encountered (88%), rendering them the most common presenting symptom in AE. Signs and symptoms in IE and AE are summarized in Table 3.

**Table 3:** Clinical symptoms of definite^c^ AE and IE patients during week 1 of hospital admission.

**Diagnostic group**	**Headache *n* (%)**	**Fever n (%)**	**Psychiatric symptoms *n* (%)**	**Cognitive symptomes *n* (%)**	**Alteration of consciousness *n* (%)**	**Movement disorders *n* (%)**	**Speech disorders *n* (%)**	**Autonomic dysfunction *n* (%)**	**Focal signs *n* (%)**	**Epileptic seizures *n* (%)**
Autoimmune encephalitis	0 (0)	2 (12)	8 (47)	6 (35)	2 (12)	2 (12)	1 (6)	4 (24)	5 (29)	15 (88)
Infectious encephalitis	19 (56)	32 (94)	28 (82)	5 (15)	19 (56)	2 (6)	8 (24)	7 (21)	10 (29)	7 (21)
*p-*value	0.0001	<0.0001	0.01		0.003					<0.0001

*Symptoms differing significantly between the two diagnostic groups are shaded in gray. P-values of these comparisons are given at the bottom of the table*.

### Diagnostic tests (condition C1; definite^c^)

All patients were investigated by cranial MRI and CSF examination, and 17/17 (AE) and 27/34 (IE) by EEG at least once during the hospital stay. Comparison of the results of paraclinical testing performed during the first week after hospital admittance revealed that increased CSF cell count was significantly more common in IE patients. On further analysis of those patients who had CSF pleocytosis (i.e., 5 or more cells/μl), the IE group displayed a significantly higher median of CSF total cells. At a criterion value of ≤ 36 cells/μl—chosen by ROC curve analysis to maximize the Youden's index—the sensitivity of diagnosis of AE was 75%, the specificity 87.5%. The rate of positive oligoclonal bands and intrathecal immunoglobulin synthesis did not differ significantly between AE and IE patients, neither did the number of patients with pathological results on cranial MRI and EEG (regional and general slowing as well as epileptic discharges were considered pathological). The most common location of supratentorial (sub)cortical MRI changes in AE patients were the mesial temporal lobes (5/17 patients), whereas extratemporal T2-alterations in IE patients were more frequent (7/34 patients) than mesio-temporal lesions (6/34 patients). Lesions in IE were predominantly localized in the thalamus and brain stem and most often found in tick-borne encephalitis. The results of the diagnostic tests are summarized in Tables 4, 5.

**Table 4:** Results of paraclinical tests of definite^c^ AE and IE patients during week 1 of hospital admission.

**Diagnostic group**	**Pleocytosis *n* (%)**	**Total CSF cell count per μl (median; range)**	**OCB positive and/or i.th. IgG-synthesis (%)**	**Pathology in EEG *n* (%)**	**Pathology on MRI *n* (%)**
Autoimmune encephalitis	8 (47)	33 (5; 200)	35	14 (82)	8 (47)
Infectious encephalitis	32 (94)	86 (7; 705)	35	23 (68)	17 (50)
*p*-value	0.0001	0.005			

*Results differing significantly between the two diagnostic groups are shaded in gray. P-values of these comparisons are given at the bottom of the table. For total CSF cell count, only those patients showing a pleocytosis (i.e., ≥ 5 cells/μl) were considered*.

**Table 5:** Localization of hyperintense lesions on T2-/FLAIR-weighted MRI performed within 1 week of hospital admission for definite^c^ AE and IE patients.

**MRI changes (FLAIR)**	**Infectious encephalitis**	**Autoimmune encephalitis**
Supratentorial extra-temporal	7	3
Latero-temporal	2	0
Mesio-temporal	6	5
Basal ganglia except thalamus	2	1
Thalamus	8	0
Cerebellum	0	0
Brain stem	7	0

*Multiple entries per patient possible*.

### Signs, symptoms, and diagnostic tests in the (probable^c^ + definite^c^) cohort

All evaluations delineated above were performed in the (probable^c^ + definite^c^) cohort as well. The main difference to the analysis of the definite^c^ cohort alone pertains to the frequency of cognitive symptoms and alterations of consciousness as presenting symptoms in AE vs. IE. The preponderance of patients presenting with cognitive symptoms in the entire AE cohort became significant at *p* = 0.03, whereas the difference concerning alteration of consciousness lost significance. Otherwise, all trends remained the same.

### Diagnostic algorithm (condition C1) – (probable^c^ + definite^c^) cohort

Among 33 AE patients (17 definite^c^ AE/16 probable^c^ AE), 19 fulfilled the criteria for possible^a^ AE according to Graus et al., 12 patients in whom well characterized autoantibodies were detected did not enter the algorithm at this point, either because they did not meet the time criterium (i.e., progression of symptoms of <3 months) or the main clinical criterium (i.e., presentation with working memory deficits, altered mental status, or psychiatric symptoms). Three patients in this group had CASPR2-antibodies, four patients had LGI1-antibodies, and one patient each had Ma2-/Ri-/GabaB-/NMDAR- and a combination of SOX1- and amphiphysin-antibodies. They either presented with subtle cognitive deficits or personality changes after several months of symptom progression or reported seizures—rather than mental deficits—as the presenting feature. The clinical features of these 12 patients are summarized in Table 6.

**Table 6:** Characteristics of the 12 definite^c^ AE patients missed by the clinical algorithm.

**Sex**	**Age (years)**	**Antibody**	**Site of antibody detection**	**Main clinical complaints**	**T0–T1 (days)**	**Follow-up (days)**	**MRI—increased FLAIR signal**	**EEG**	**CSF—Pleocyt. (cells/μl)**	**Tumor**
Male	57	CASPR2	Serum, CSF	Cognitive symptoms, seizures	180	1,077	Left amygdala	Left temporal epileptic discharges	Yes (7)	No
Male	30	Ma2	Serum, CSF	Cognitive symptoms, psychiatric alterations, seizures	150	987	Left temporomesial	Epileptic discharges	Yes (5)	No
Female	57	Ri	Serum	Opsoclonus	56	638	Microangiopathy	Not done	Yes (64)	Cancer of unknown origin
Male	73	LGI1	Serum	Seizures, autonomic symptoms	49	1,619	Right (para-) hippocampus	Epileptic discharges	No	Prostate carcinoma
Male	65	GABA B	Serum	Seizures, facial palsy	14	350	Normal	General slowing	No	No
Female	67	LGI1	Serum, CSF	Seizures	8	920	Right amygdala and hippocampus	General slowing	Yes (11)	No
Male	40	NMDAR	Serum, CSF	Seizures, dysarthria, dysphagia	7	385	Bilateral (sub) cortical	Epileptic discharges	No	No
Male	72	CASPR2	Serum, CSF	Seizures	150	427	Right hippocampus	Regional slowing	No	No
Male	65	LGI1	Serum	Seizures	21	267	Microangiopathy	Normal	No	No
Female	70	SOX1, Amphiphysin	Serum, CSF	Seizures, hemiparesis	10	881	Normal	Epileptic discharges	No	Neuroendocrine bronchial carcinoma
Male	64	CASPR2	Serum, CSF	Seizures	1	2,321	Normal	Normal	No	No
Female	39	LGI1	Serum	Cognitive symptoms, psychiatric alterations, seizures	270	1,431	Normal	Not done	No	No

Three of the remaining 19 AE patients qualified for the category definite^a^ autoimmune limbic encephalitis due to quasi-pathognomonic bilateral, strictly mesial changes of the temporal lobe on T2-/FLAIR (fluid-attenuated inversion recovery)-weighted MRI or bilateral, mesio-temporal hypermetabolism on FDG-PET as an alternative imaging criterium approveded by Graus et al. for the diagnosis of definite^a^ limbic encephalitis. All three cases (3 females, age 29–61 years) were autoantibody-negative.

One patient with the clinical diagnosis of ADEM fell into the probable^a^ autoimmune category as the definite diagnosis of ADEM according to the algorithm would have required the absence of new clinical or MRI findings 3 months after symptom onset. Hence, this diagnosis cannot be made during the first week by definition.

3 patients fulfilled the criteria of clinical NMDARE, therefore being considered probable^a^ autoimmune. In one of these NMDAR-antibodies could be detected, the other two patients were finally considered as autoantibody-negative AE (both tested negative for NMDAR-antibodies on 1 and 5 occasions, respectively). Four oligosymptomatic NMDAR-antibody-positive patients did not exhibit the minimum number of major symptom groups during the first week of hospital admission and therefore remained in the possible^a^ AE category.

Two patients diagnosed with probable Bickerstaff encephalitis did not enter the algorithm as they failed the clinical criteria of possible^a^ AE due to lack of cognitive, mental or psychiatric symptoms. They exclusively presented with ataxia and central oculomotor symptoms. GM1, GD1b and GD1a antibodies—but no GQ1b antibodies—were detected in both patients' serum.

The categories “cell-surface/onconeuronal antibodies” and “thyroid antibodies” were not considered at this point as per study design. Finally, two patients with the clinical diagnosis of antibody-negative AE actually fulfilled the corresponding criteria of the algorithm, leaving a total of 10 AE patients in the “reconsider diagnosis” category.

Almost all IE patients (92%) qualified for the diagnosis of possible^a^ AE under the premise that the results of microbiological testing were unknown. Four fulfilled the criteria for “clinical NMDARE—probable^a^ AE” and 13 for “antibody-negative AE.”

Our analysis resulted in a sensitivity of 58% and a specificity of 8% for the “possible^a^ AE” category during the first week of admission under the assumption of ignorance of the autoantibody status/ microbiological test results, corresponding to a PPV of 29% and a NPV of 22%. The category “clinical NMDARE—probable^a^ AE” resulted in a sensitivity of 20% and a specificity of 92% (PPV = 14%, NPV = 95%), the category “definite^a^ limbic AE” in a sensitivity of 13% and a specificity of 100% (the 23 AE patients not diagnosed with parainfectious encephalitis/ADEM, NMDARE or Bickerstaff encephalitis were considered as limbic encephalitis). In total, 9 (1/8) of all AE patients were diagnosed as probable^a^ or definite^a^ AE under condition C1, corresponding to a sensitivity of 27%.

### Diagnostic algorithm (condition C1) – definite^c^ cohort

Among 17 definite^c^ AE, 5 fulfilled the criteria for possible^a^ AE according to Graus et al., 1 patient fulfilled the criteria of clinical NMDARE, therefore being considered probable^a^ autoimmune. None of the remaining patients fulfilled the criteria of antibody-negative AE.

Almost all IE patients (94%) qualified for the diagnosis of possible^a^ AE under the premise that the results of microbiological testing were unknown. Three fulfilled the criteria for “clinical NMDARE—probable^a^ AE.” They were diagnosed with HSV1 encephalitis (2 patients) and TBE (1 patient). Ten patients fulfilled the criteria for “antibody-negative AE” (HSV1 encephalitis in 2 patients, TBE in 8 patients).

The subanalysis of the definite^c^ group of patients only rendered a sensitivity of 29% and a specificity of 6% for the “possible^a^ AE” category during the first week of admission under the assumption of ignorance of the autoantibody status/ microbiological test results, corresponding to a PPV of 14% and a NPV of 14%. The category “clinical NMDARE - probable^a^ AE” resulted in a sensitivity of 20% and a specificity of 91% (PPV = 25%, NPV = 91%). In total, 1 of all definite^c^ AE patients was diagnosed as probable^a^ AE under condition C1, corresponding to a sensitivity of 6%.

### Diagnostic algorithm (condition C2) – (probable^c^ + definite^c^) cohort

**Figure 1:**
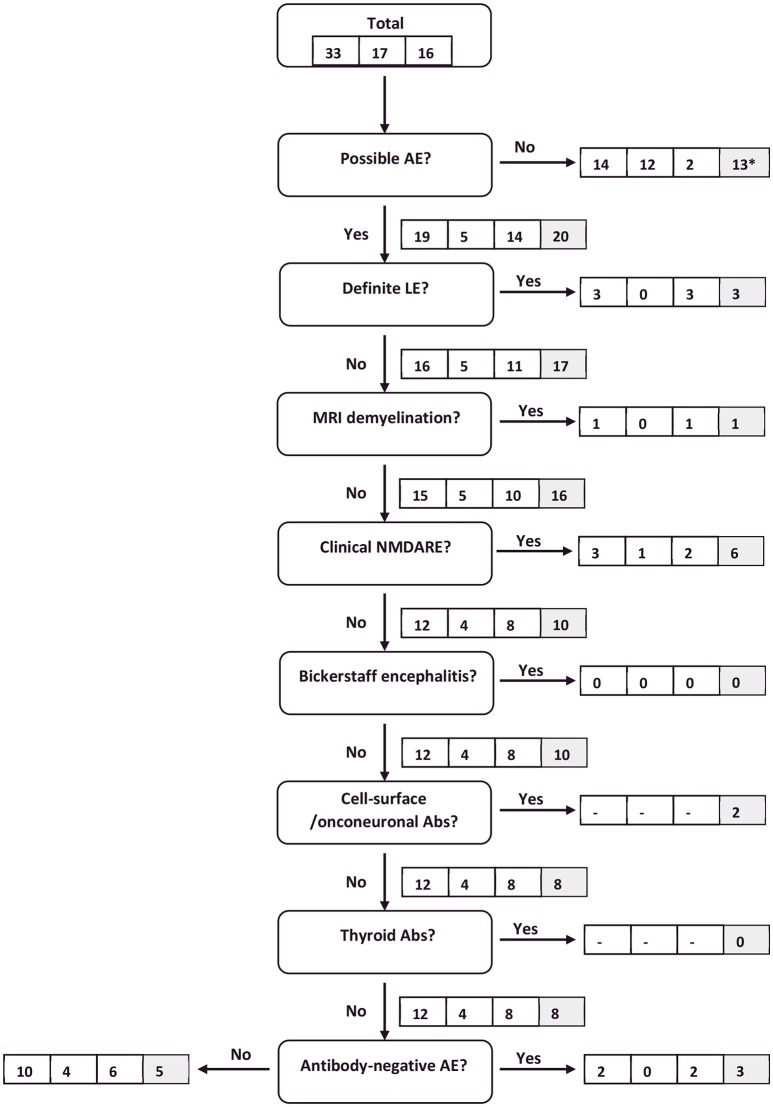
Flow chart of AE patients following the clinical algorithm suggested by Graus et al. (22). Framed figures adhere to the following sequence: all AE patients/definite^c^ AE patients/probable^c^ AE patients (condition C1). Where applicable, the last box (shaded in gray) provides the respective figure for all AE patients under condition C2. ^*^11 antibody-positive patients are included in this number. They would eventually have been diagnosed with AE based on antibody-status. However, as antibody-status does not feature in the “possible AE” criteria, they were excluded at this point.

**Figure 2:**
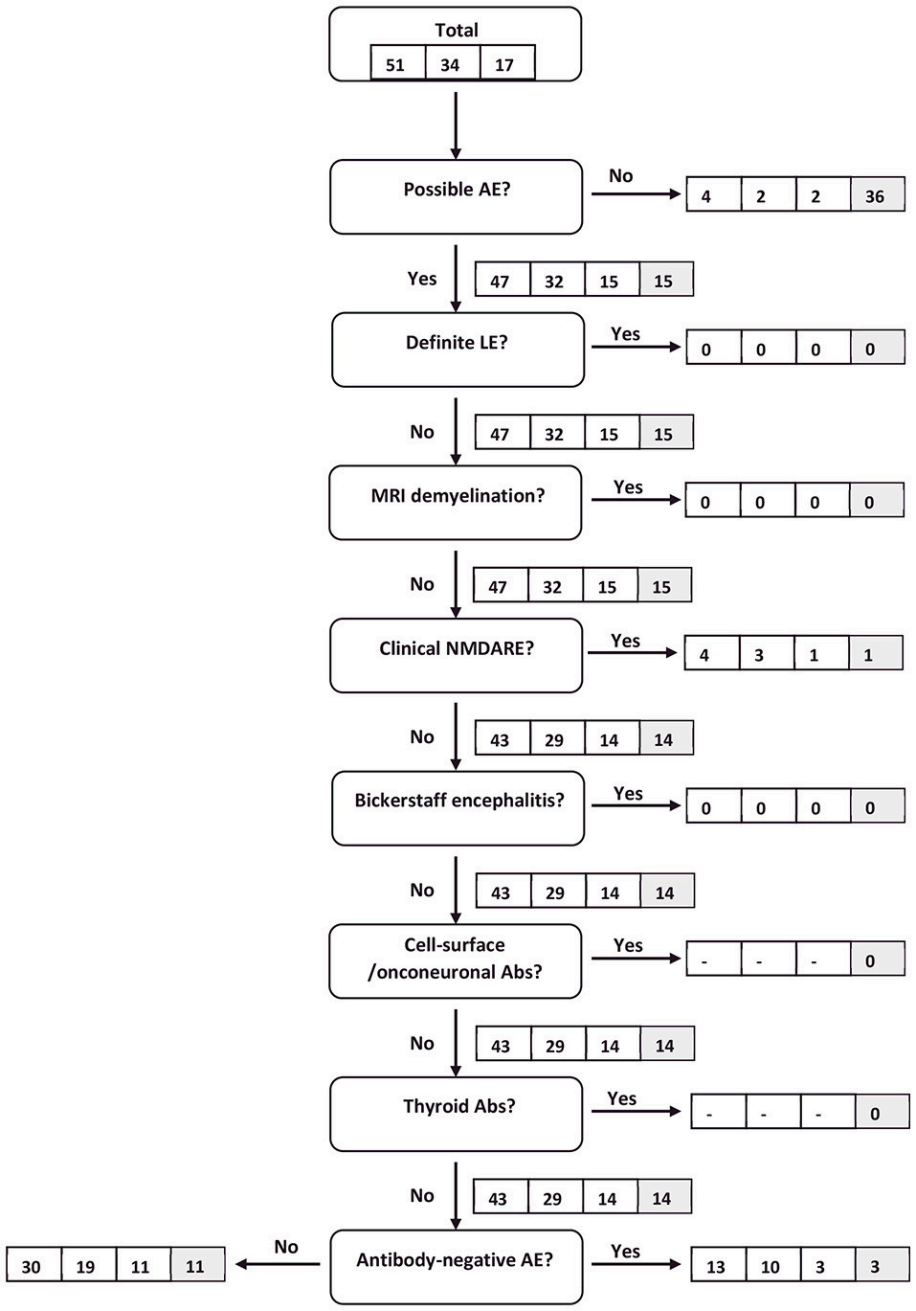
Flow chart of IE patients following the clinical algorithm suggested by Graus et al. (22). Framed figures adhere to the following sequence: all IE patients/definite^c^ IE patients/probable^c^ IE patients (condition C1). Where applicable, the last box (shaded in gray) provides the respective figure for all IE patients under condition C2.

Permitting all clinical information obtained for each patient—including test results from autoantibody and microbiological testing—to be taken into consideration, 36 IE patients were excluded from the algorithm by the “reasonable exclusion of alternative causes” criterium. Hence, the specificity of “possible^a^ AE” increased to 71%. The sensitivity of this criterion changed only marginally (58–61%), whereas the sensitivity for the diagnosis of all probable^a^ or definite^a^ AE increased from 27 to 45% and for “clinical NMDARE - probable^a^ AE” from 20 to 80%. The latter increase was due to three NMDARE patients developing one or more major symptoms after 3, 4, and 6 weeks. One NMDARE patient did not meet the “possible^a^ AE” criteria due to lack of mental/psychiatric symptoms and would therefore not have been considered in the NMDARE category when strictly following the algorithm in a successive fashion. The NMDARE diagnostic panel applied in isolation would have resulted in a sensitivity of 100% under condition C2. For a graphic illustration of the application of the clinical algorithm see Figures 1, 2.

### Diagnostic algorithm (condition C2) – definite^c^ cohort

With all clinical information taken into consideration, all 34 definite^c^ IE patients were excluded from the algorithm by the “reasonable exclusion of alternative causes” criterium. Hence, the specificity of “possible^a^ AE” increased to 76%. The sensitivity of this criterion was 35%. All 6 patients diagnosed as possible^a^ AE went on to be diagnosed as probable^a^ or definite^a^ AE due to their positive antibody status. The sensitivity for “clinical NMDARE - probable^a^ AE” remained at 80%.

## Discussion

The differential diagnosis of IE and AE is notoriously difficult, particularly at an early stage after symptom onset. Hence, the first aim of our analysis was to define a subset of presenting symptoms and paraclinical test results in order to facilitate distinguishing between these two entities. We found that they differed significantly in respect to epileptic seizures, fever, headache, psychiatric symptoms, alteration of consciousness, and CSF pleocytosis during the first week of hospital admission.

In concordance with our results, previous studies found seizures to be less frequent in IE than in AE individuals (13, 26, 27). Fever and headache have been reported to occur more often in IE patients (13). Another study showed mixed results though, with fever being less common in AE individuals than in patients with HSV1 encephalitis, but slightly more frequent than in patients with VZV encephalitis (27). The same group reported headache to be slightly more common in AE than in HSV1 encephalitis, but less frequent than in VZV encephalitis. These inconsistencies between different studies and our results are most likely due to the heterogeneity of the pathogenic agents included in the analyses. They may also result from our focus on the symptoms at the time of a patient's initial presentation rather than on all symptoms during the entire course of the disease.

Whereas alterations of consciousness are common to both IE and AE patients (13, 26), these former studies revealed a higher incidence of psychiatric symptoms in AE, seemingly contradicting our results. This is most likely due to our wide definition of psychiatric symptoms, including psychomotor slowing and lethargy. Previous reports have shown the latter to be frequent symptoms in AE as well as in IE (27).

As to the paraclinical tests, previous publications support our claim that CSF pleocytosis is less frequent and milder in AE than in IE. Comparing a cohort of NMDARE with IE patients, Gable et al. reported a higher median cell count in those with IE for most infectious pathogens with the exception of rabies (13, 26). Their findings as to the prevalence of MRI changes in AE patients (46%) closely resemble our results as well (13). The rate of MRI pathologies in IE reported by this group ranges from 40 to 100%, dependent on the specific pathogen. The majority of their patients displayed some form of EEG pathology (AE and IE), again confirming our findings. The relatively low percentage of oligoclonal bands/intrathecal immunoglobulin synthesis in our cohort is most likely due to us reporting the CSF analysis performed during the first week of admission, when intrathecal immunoglobulin production may not yet have started.

The next step of our approach was to apply the Graus algorithm to encephalitis patients under the condition C1. We tested the definite^c^ patients alone as well as the (probable^c^ + definite^c^) patients to obtain both the best scientific rigor possible as well as the conclusions drawn from a larger and more heterogeneous cohort, including clinical pictures considered in the algorithm that frequently (ADEM in the adult) or always (antibody-negative encephalitis) lack definite confirmation by immunological testing. Both sensitivity and specificity were low or very low for the diagnosis of “possible^a^ AE,” “clinical NMDARE - probable^a^ AE,” and AE of all levels of diagnostic confidence for both the definite^c^ and the (probable^c^ + definite^c^) cohort. The respective sensitivity increased under the condition C2, particularly so for “clinical NMDARE - probable^a^ AE.” The same is true for the specificity, mainly due to the exclusion of IE cases with positive microbiological testing in the first step of the algorithm.

In an approach similar to ours, the algorithm was evaluated by a Chinese group on 95 patients, 64 of whom had AE (28). The remaining 31 cases included viral encephalitis (14 cases), purulent encephalitis (2), tuberculous meningoencephalitis (2), CNS tumor (3), and epileptic disorders (10). Their selection with a ratio of only 45% viral IE is most likely partially responsible for their much higher specificity of the “possible^a^ AE” diagnosis (83% at days 0–14 of admission, increasing to 92% afterwards), as viral encephalitis seems to be the most difficult to be distinguished from AE compared to encephalopathies of other origin. The overall sensitivity reported by Li et al. for possible^a^ AE was higher (84%) than the one we calculated in our entire collective either under condition C1 or C2. This is probably due to a higher ratio of NMDARE cases (61% of all AE) in their collective, for which the algorithm seems to have a particularly high sensitivity (29). Notably, the sensitivity reported by Li et al. for possible^a^ AE, definite^a^ limbic AE, and probable^a^ NMDARE for the time period of up to 14 days after admission very closely resembles our data for condition C1 in the entire patient group: 60 vs. 58% (possible^a^ AE), 10 vs. 13% (definite^a^ limbic AE), and 16 vs. 20% (probable^a^ NMDARE).

In an Australian cohort of 29 children with NMDARE, the authors found a time-dependent sensitivity of the Graus diagnostic criteria for “clinical NMDARE - probable^a^ AE” of 24% after 1 week of symptoms, rising to 90% when the entire time of inpatient hospital admission was taken into account (29). The median time to fulfilling the diagnostic criteria was 2 weeks. Three children with IE (enterovirus, mycoplasma) fulfilled the criteria as well, again demonstrating the difficulties in delimiting AE from non-granulocytic IE.

Both studies and our own data confirm that the sensitivity of the clinical algorithm for the diagnosis of AE is clearly time-dependent, restricting its usefulness in the acute setting. However, it remains a valuable diagnostic aid for antibody-negative AE or in resource-poor settings, where access to advanced serological diagnostics is limited. Furthermore, the specificity of “clinical NMDARE - probable^a^ AE” and “definite^a^ limbic AE” is high, encouraging the initiation of immunosuppressive therapy if the respective criteria apply – even in the absence of serological proof. Considering the low sensitivity at initial presentation and the importance of early therapy, however, therapy should not be withheld until all criteria are fulfilled but rather started if an AE is deemed likely (29).

CASPR2- and LGI1-antibody associated encephalitis poses a particular diagnostic challenge. Not only are CSF and MRI unremarkable in many cases, these were also the AE to most frequently escape detection by the clinical algorithm due to their often subtle evolution. Furthermore, commercially available cell-based assays used for antibody detection seem to have the lowest sensitivity for CSF CASPR2- and LGI1-antibodies when compared to anti-GABA_B_, -GAD65, and -NMDAR (30). The sensitivity was higher when testing the serum, although this may introduce more unspecific results. These findings should motivate to persist with the diagnostics—i.e., involve a research laboratory for further serological testing—when the clinical suspicion of anti-CASPR2-/LGI1-encephalitis remains high despite negative diagnostics.

An interesting secondary finding pertains to the 3 patients diagnosed as “definite limbic encephalitis” on the grounds of the Graus algorithm. All of them were antibody-negative, young to middle-aged (29–61 years) women who presented with epileptic seizures (refractory epileptic status in one patient). Epidemiologically, this cohort resembles previous patient groups diagnosed with NORSE [new-onset refractory status epilepticus; see for example (31)]. However, they significantly differ from an antibody-negative AE cohort recently described, which mainly consisted of elderly males presenting with short-memory loss (32). These divergent findings insinuate that the manifestation of antibody-negative AE comprises distinct pathologies.

On the part of IE, TBE was particularly difficult to distinguish from AE. Albeit very sensitive, there may be pitfalls associated with the specific serology if TBE-virus antibodies are determined very early during the course of the disease, due to cross-reactivity with other flavivirus or to previous TBE-vaccination (33, 34). The encephalitic form of TBE virus infection often goes along with psychiatric symptoms such as psychomotor slowing and decreased vigilance. In some cases, speech disorders, epileptic seizures and/or movement disorders occur, rendering the clinical picture similar to NMDARE. Furthermore, TBE patients frequently show bilateral basal ganglia/thalamic involvement, fulfilling the Graus criteria of “MRI features suggestive of encephalitis,” which they define as “brain MRI hyperintense signal on T2-weighted fluid-attenuated inversion recovery sequences […] in multifocal areas involving gray matter, white matter, or both compatible with demyelination or inflammation” (22). If the serology is equivocal, TBE may therefore easily be mistaken for autoantibody-negative AE as reflected by the high proportion of TBE-patients in this group under condition C1. It would be interesting to investigate whether this is true for other flaviviruses as well. The high prevalence of TBE in our sample certainly contributed further to the low specificity of the algorithm.

Limitations of our study include that not all IE patients were investigated with the immunological panel. This is particularly relevant in the light of recent discoveries of AE being triggered by viral infection, such as post-HSV-encephalitis NMDARE (35). Similar restrictions apply to AE patients: although all of them were tested for antineuronal antibodies, the extent of the panels varied according to the respective knowledge at the time of testing. We attempted to overcome these limitations by conducting all analyses not only on the entire cohort, but also on the subgroup of those patients, in whom definite diagnoses had been possible. The conclusions pertaining to presenting clinical signs, symptoms and paraclinical test results were very similar in both groups. The problem of a low sensitivity and specificity of the Graus algorithm was more pronounced in the subgroup containing only patients with positive immunological/microbiological test results.

As to the survey of presenting symptoms, cognitive symptoms may have been underestimated in both diagnostic groups for difficulty of assessment in patients suffering from psychiatric symptoms or altered consciousness. Furthermore, mild psychiatric or cognitive changes may have been underdiagnosed if underreported by the patient and his family and unrecognized by the physician at admission. Further shortcomings result from the heterogeneity of AE and IE aetiologies in our rather small cohort as well as the retrospective nature of the study. Prospective larger investigations are warranted to further explore the intricate challenge of early diagnosis in encephalitis.

## Author contributions

JW conceived the study design, is responsible for statistical analysis and data interpretation, and wrote the manuscript. OK, MS, IK, and TvO helped with data acquisition and interpretation and critically revised the manuscript.

### Conflict of interest statement

JW reports personal fees from UCB Pharma GmbH and travel funds from Boehringer Ingelheim GmbH and Daiichi Sankyo GmbH. TvO reports personal fees and non-financial support from Eisai Pharma GmbH Vienna, grants, personal fees and non-financial support from UCB Pharma GmbH Vienne, non-financial support from Medtronic Österreich GmbH, grants, personal fees and non-financial support from Novartis Pharma, personal fees from Roche Pharma, personal fees from Biogen Idec Austria, personal fees from Liva Nova, personal fees from Sanofi Aventis GmbH, and grants from Grossegger & Drbal GmbH. The remaining authors declare that the research was conducted in the absence of any commercial or financial relationships that could be construed as a potential conflict of interest. The reviewer CB declared a past co-authorship with one of the authors TvO to the handling Editor.
