# Supplementary material for: Evaluation of Clinical and Paraclinical Findings for the Differential Diagnosis of Autoimmune and Infectious Encephalitis
Source: Front Neurol. 2018 Jun 8;9:434. doi: 10.3389/fneur.2018.00434 (PMC6008545; doi:10.3389/fneur.2018.00434)
Supplement: Supplementary file 1 [file Table_1.DOCX]

Supplementary Material

**Evaluation of clinical and paraclinical findings for the differential diagnosis of autoimmune and infectious encephalitis**

**Wagner JN*, Kalev O, Sonnberger M, Krehan I, von Oertzen TJ**

*** Correspondence:**

Judith N Wagner, MD

Kepler University Hospital, Department of Neurology 1

Wagner-Jauregg-Weg 15, 4020 Linz, Austria

Tel. +43 (0)5 7680 87 – 25724, Fax +43 (0)5 7680 87 - 45701

[Judith.Wagner@kepleruniklinikum.at](mailto:Judith.Wagner@kepleruniklinikum.at)

ORCID 0000-0002-0776-6821

# Supplementary Table

**Supplementary Table 1.** The table shows the extent of the antibody panels used during the time period relevant for the study as well as the number of AE patients investigated with the respective panel. Only the last (and hence most complete) panel each patient was investigated with during initial diagnosis and/ or follow-up was counted (thus there are no entries for 2007-2009). 11/51 IE patients received screening for autoantibodies (1 pt 2009, 1 pt 2011, 2 pts 2013, 1 pt 2015, 2 pts 2016, 4 pts 2017). Ganglioside antibody panels included GM1/2, GQ1b, GD1a/b and GT1b/GM3 (until 2015). Anti-MAG-antibodies were included in 2015. Only the subset of patients suspected to have Bickerstaff encephalitis were investigated with the ganglioside panels.

|  |  | **2009** | **2010** | **2011** | **2012** | **2013** | **2014** | **2015** | **2016** | **2017** |
| --- | --- | --- | --- | --- | --- | --- | --- | --- | --- | --- |
| **Intracellular antigens** | Hu | x | x | x | x | x | x | x | x | x |
|  | Yo | x | x | x | x | x | x | x | x | x |
|  | Ri | x | x | x | x | x | x | x | x | x |
|  | Amphiphysin | x | x | x | x | x | x | x | x | x |
|  | Ma2/PNMA2 | x | x | x | x | x | x | x | x | x |
|  | CV2 | x | x | x | x | x | x | x | x | x |
|  | PCA2 |  |  | x | x | x | x | x | x | x |
|  | Anti-glial nuclear abs/SOX1 |  |  | x | x | x | x | x | x | x |
|  | Recoverin |  |  |  |  | x | x | x | x | x |
|  | Zic4 |  |  |  |  |  |  | x | x | x |
|  | TR |  |  |  |  |  |  | x | x | x |
|  | GAD |  |  |  |  |  |  | x | x | x |
| **Extracellular antigens** | NMDAR |  |  | x | x | x | x | x | x | x |
|  | CASPR2 |  |  | x | x | x | x | x | x | x |
|  | LGI1 |  |  | x | x | x | x | x | x | x |
|  | AMPA |  |  | x | x | x | x | x | x | x |
|  | GABAb |  |  | x | x | x | x | x | x | x |
|  | DPPX |  |  |  |  |  |  |  |  | x |
| **No of AE patients** |  | 2 | 1 | 1 | 1 | 1 | 3 | 4 | 8 | 12 |
